# Risk of Colorectal Cancer among Patients with One or Multiple Metabolic Syndrome Components

**DOI:** 10.3390/cancers16193350

**Published:** 2024-09-30

**Authors:** Shanmuga Sundaram, Rajan Lamichhane, Alfred Cecchetti, Usha Murughiyan, Uma Sundaram

**Affiliations:** 1 Department of Clinical and Translational Sciences, Joan C. Edwards School of Medicine, Marshall University, 1600 Medical Center Drive, Huntington, WV 25701, USA; 2 Department of Internal Medicine, Joan C. Edwards School of Medicine, Marshall University, 1600 Medical Center Drive, Huntington, WV 25701, USA

**Keywords:** metabolic syndrome, colorectal cancer, screening, obesity, insulin resistance, low high-density lipoprotein cholesterol, hypertension, hypertriglyceridemia, risk factors, age

## Abstract

**Simple Summary:**

This research identifies the critical metabolic syndrome components that lead to a higher risk of developing colorectal cancer. This is a crucial step in creating colon cancer screening guidelines based on the presence of some specific MetS components or combinations of components. This pivotal insight could inform future CRC screening strategies. Finally, our findings could fill the knowledge gaps in current research areas relevant to metabolic syndrome and colorectal cancer.

**Abstract:**

**Background/Objectives**: Dysfunctions of metabolic syndrome (MetS) have been identified as a significant risk factor for colorectal cancer (CRC). However, current colon cancer guidelines do not classify patients with MetS as high risk, thereby leaving these individuals vulnerable. Consequently, we explored the relationship between MetS, its individual components, and the development of CRC in a cohort of patients with MetS to assess the necessity for CRC screening in these individuals. **Methods**: This study included patients ages 18 and older that received a service from the Marshall-Health (MH) practice plan, Cabell-Huntington Hospital (CHH), MU/JCESOM’s Edwards Comprehensive Cancer Center (ECCC), or the University of Kentucky HealthCare (UKHC) system between 2010 and 2018. We implemented log-binomial regression models to assess the individual and collective effects of MetS components after adjusting other CRC risk factors. **Results**: Given that CRC prevalence increases in the older population (aged 65 years and above), and that multiple components of MetS are observed within the same population, we analyzed the concurrent impact of all MetS components on CRC. Log-binomial regression models were implemented to assess the risk of CRC due to MetS components after adjusting other risk factors. **Conclusions**: We identified specific components that markedly increased CRC risk, suggesting that individuals with these components should be prioritized for early screening. These findings could significantly influence early CRC screening protocols, with the ultimate aim to reduce mortality associated with the disease.

## 1. Introduction

**Figure 1:**
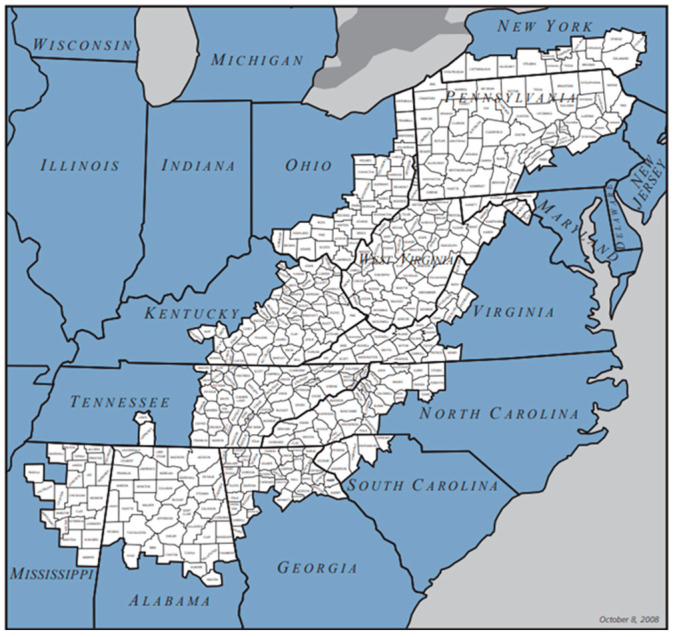
Map of Appalachian region [21].

Colorectal cancer (CRC) remains one of the most treatable forms of cancer, yet it stands as the third leading cause of cancer-related mortality among both men and women in the United States, as well as the second leading cause when considering both genders combined. According to the American Cancer Society, it was responsible for an estimated 53,200 deaths in 2020; the age-adjusted mortality rate was 12.5 per 100,000 population [1]. Metabolic dysfunction associated with metabolic syndrome (MetS) has been linked to a variety of adverse health outcomes, including type 2 diabetes (T2DM), cardiovascular disease (CVD), liver disease, chronic kidney disease, and several types of cancer [2,3,4,5,6,7]. MetS includes five key components: insulin resistance (IR), hypertension (HTN), hypertriglyceridemia (HG), low high-density lipoprotein cholesterol (HDL), and obesity. Even though several studies have been conducted on the relationship between MetS and CRC [7,8,9,10,11,12,13,14,15], there has been limited focus on the individual components of MetS, except obesity. Components such as obesity, dyslipidemia, and impaired glucose tolerance have been linked to increased risk for CRC [8,16], but many studies have not controlled for other CRC risk factors. Given the higher prevalence of CRC among the elderly—who are also more likely to suffer from multiple MetS components—it is critical to study the combined effects of these components on CRC risk. Understanding the impact of each MetS component is vital for developing targeted CRC screening strategies. In our study, we aimed to identify which components or combinations thereof are most strongly associated with an increased risk of developing CRC. This research specifically focused on the Appalachian population, which suffers from higher rates of both colon and rectal cancers and associated mortality rates across both genders [17]. Appalachia, a region extending over 13 states and home to about 25 million people, or 8.2% of the U.S. population, is largely rural, with approximately 42% of its population living in areas defined as rural [18]. Socioeconomic status and healthcare availability are critical to overall health and well-being and are often key determinants of health. The Appalachian region generally experiences lower economic status, lower educational attainment, and reduced access to healthcare compared to the general U.S. population. As expected, this contributes to poorer health outcomes, including elevated CRC incidence. This study is particularly important for understanding the mechanisms of CRC in a medically underserved rural population. Another contributing factor to the high CRC mortality rate in Appalachia is the low rate of cancer screenings, particularly in rural areas, compared to the national average [19,20]. Despite the significant risk posed by MetS for CRC, current guidelines do not categorize MetS patients as high risk, typically advising only caution, thus exposing them. It is thus crucial to understand the role of each MetS component in developing targeted CRC screening and prevention strategies. By exploring the relationship between MetS and its components and CRC, this study aimed to determine the necessity for CRC screening among those with MetS and/or specific MetS components. The recommendations elucidated from this study will help formulate guidelines and strategies for mitigating CRC risk focused on MetS components. This will benefit not only the Appalachian region shown in Figure 1 but potentially the entire nation.

## 2. Materials and Methods

This study included patients ages 18 and older who received a service from the Marshall-Health (MH) practice plan, Cabell-Huntington Hospital (CHH), MU/JCESOM’s Edwards Comprehensive Cancer Center (ECCC), or the University of Kentucky HealthCare (UKHC) system between 2010 and 2018. The study population was primarily located in Central and North Central Appalachia, which includes the western part of West Virginia, the southern part of Ohio, and the eastern part of Kentucky. Patients were classified as having metabolic syndrome if they met at least 3 of the following 5 conditions:

- Insulin resistance (IR): average fasting glucose ≥100 mg/dL, HbA1c >= 5.7 at any point, receiving drug therapy for hyperglycemia, or type 2 diabetes listed as a billing diagnosis or under the problem list.
- Hypertension (HTN): blood pressure ≥130/85 mm Hg, receiving drug therapy for hypertension, hypertension listed as a billing diagnosis or under the problem list.
- Hypertriglyceridemia (HG): average triglycerides ≥150 mg/dL, receiving drug therapy for hypertriglyceridemia, or hypertriglyceridemia listed as a billing diagnosis or under the problem list.
- Low high-density lipoprotein cholesterol (HDL-C): average HDL-C < 40 mg/dL in men or <50 mg/dL in women, receiving drug therapy for reduced HDL-C, or low HDL-C listed as a billing diagnosis or under the problem list.
- Obesity: average BMI > 30, or obesity listed as a billing diagnosis or under the problem list.

Only 263,023 patients with available data to determine the presence or absence of all 5 conditions were included in this study. The patient data relevant to the study were extracted from Marshall University’s Appalachian Clinical and Translational Science Institute (ACTSI) Clinical Data Warehouse and UKHC’s Enterprise Data Warehouse. The study variables are listed below:

1. Demographic variables—current age and gender.
2. Body Mass Index (BMI) (in k Lamichhane, g/m^2^)—Current BMI. We chose BMI because waist circumference was not available in the data.
3. Socioeconomic status (SES)—Insurance was used as a proxy for SES, as patient income and education information was unavailable. We accounted for two categories of insurance: Medicaid and Others or unknown, which also included self-pay. Due to a lack of information on different insurance types, we could not further break down the insurance categories.
4. Family history of colorectal cancer (CRC)—patients with a family history of CRC were identified using diagnosis codes (ICD9 or ICD10), SNOMED codes, and problem lists.
5. Social history (tobacco and alcohol use history)—If the use of any tobacco, including smoking, or alcohol was ever documented during the study period, the patient was said to have a history of tobacco and/or alcohol use. Conversely, if the patient had a documented history of never using tobacco and/or alcohol, they were said to have no history of tobacco and/or alcohol use, respectively.
6. Inherited syndromes—whether the patient had a history of familial adenomatous polyposis, hereditary non-polyposis colorectal cancer, Lynch Syndrome, Turcot Syndrome, or Peutz–Jeghers Syndrome.
7. History of polyps.
8. History of inflammatory bowel disease.
9. Presence or absence of insulin resistance, hypertension, low HDL-C, hypertriglyceridemia, and obesity.
10. The number of MetS criteria met by the patient.
11. Metabolic syndrome (MetS)—patients who met 3 or more of the 5 conditions mentioned above were classified as MetS patients. In contrast, those who did not meet at least 3 criteria were classified as non-MetS patients.
12. Presence or absence of colorectal cancer.
13. Individual MetS components—IR, low HDL, HTN, and HG.

### Statistical Analyses

Data from the individuals who reported all the components of MetS were included in this study. Data from ACTSI and UKHC were combined based on common variables mentioned in ICD9/ICD10 codes, billing diagnoses, and reported problem lists. Descriptive summaries were presented as mean ± SE for the continuous variables and frequency (percent) ± SE for the categorical variables. Relative risk and attributable risk of CRC among individuals who met the criteria for MetS and other components were individually calculated. We investigated how MetS, IR, low HDL, HTN, and HG individually impact the risk of CRC by using log-binomial regression models after adjusting for other risk factors (covariates) of CRC. As multiple components co-occur, it is important to investigate the simultaneous effect of all these components on CRC. We implemented a log-binomial regression of CRC on IR, low HDL, HTN, and HG, taken together, by adjusting other risk factors. This clarified which components of MetS contributed to the higher risk of CRC when taken collectively. Once the important components were identified, we compared the risk of CRC among the individuals who had those components and their combinations against that of those who had the other components. We wanted to compare the risks among patients aged <50 yrs., 50–65 yrs., and ≥65 yrs., but there were not enough CRC cases among patients <50 yrs. old, so we divided the population into two groups of those <65 yrs. and those ≥65 yrs. old for analyses. In our analyses, colorectal cancer status, Yes/No, was the outcome variable, while MetS status; each of the MetS components, IR, HTN, HG, and HDL; and combinations of significant important components obtained previously, taken separately, were the primary predictor variables. In the analyses, we controlled the other variables, demographic—age, gender, and BMI; socioeconomic status—insurance status as a proxy of SES; social behavior—history of tobacco use and alcohol use; history of risk factors—history of polyps, inflammatory bowel disease, inherited syndromes or family history of CRC; and type II diabetes status (T2DM), to account for the effect of these variables on CRC status. These control variables were selected as they are some of the identified risk factors of colorectal cancer [22,23,24,25,26,27,28,29]. Obesity and other components of MetS, to some extent, may represent a shared causal mechanism in the development of CRC. We did not directly relate obesity with CRC; instead, we used BMI as one of the risk factors and controlled for it in the model. As the links between MetS and T2DM and IR and T2DM are well studied, and both MetS and IR are the most significant predictors of the development of T2DM [2,3,6,7,30], we removed T2DM as one of the covariates for the analyses where MetS, IR, and their combinations, taken separately, were primary predictors in order to avoid multicollinearity and overfitting.

All statistical analyses were performed using SAS version 9.4 (SAS Institute, Cary, NC, USA). Results were also validated by cross-checking with other software, R (version 3.6.3). The results were considered statistically significant when the *p*-values were less than 0.05.

## 3. Results

Data from ACTSI and UKHC data warehouses of individuals with all MetS components reported between 2010 and 2018 were included in the analyses. Table 1 presents the distribution of demographic and other controlled variables together with the individual MetS components of 263,023 individuals. In the study population, the median age was 61 years, while the average BMI was 30.9, which indicated a large overweight and obese population. Of the study population, 47.7% of individuals were obese and 29.9% were overweight; this weight status reflects the obesity and overweight status of the elderly Appalachian population. There were more females (53.4%) than males (46.6%). In terms of age, 26.1% of individuals were <50 yrs., 32.1% were between 50 and 65 yrs. old, and 41.8% were more than 65 yrs. old. Metabolic syndrome criteria were met by 80.4% of patients; similarly, IR, HDL, HTN, and HG criteria were met by 73.9%, 80.7%, 80.3%, and 77.7% of patients, respectively. In the study population, 48.8% had some history of tobacco use, 11.9% were alcohol users, 6.7% had a history of CRC or any inherited syndromes, 5.9% had a history of IBD, 8.1% had polyps, and 36.6% were diabetic.

Table 2 presents the incidence risk ratio of CRC among the patients with different components of MetS. MetS and all components of MetS led to a significantly higher risk of CRC. It is noticeable that the risk ratio of CRC is high among the patients who had IR and met the MetS criteria. For patients who met the MetS criteria, the risk of CRC was 1.97 times compared to those who did not. Patients who had IR were at 2.1 times higher risk to develop CRC than those who did not have IR. Similarly, risk ratios for HDL, HTN, and HG were 1.8, 1.87, and 1.85. Among patients who met MetS criteria, the portion of the incidence of developing CRC that could be attributed to IR was 72 per 10,000. Similarly, there were 79, 64, 68, and 68 new CRC cases for every 10,000 patients who had IR, low HDL, HTN, and HG, respectively.

The individual effects of MetS components on CRC were assessed by adjusting demographics, SES, and other variables considered significant in determining CRC. We used log-binomial regression of CRC on MetS and its components after adjusting age, BMI, sex, insurance information, smoking habits, alcohol consumption, family history of CRC or inherited syndromes, history of inflammatory bowel disease, history of polyps, and type II diabetes. Table 3 shows the individual effects of MetS, IR, HDL, HTN, and HG on CRC, taken separately, after adjusting the above-mentioned risk factors. The risk of CRC among patients who met MetS criteria was 28% higher than among patients who did not meet the MetS criteria. We observed the highest risk of CRC in patients with IR; the risk of CRC among patients with IR was 1.60 times the risk of CRC among those who didn’t have IR. Similarly, the risk of CRC among individuals who had low HDL, high HTN, and high HG were 1.29, 1.13, and 1.17 times compared to those who did not show these symptoms.

The risk for individual components was low since the same individual could have multiple components, and by considering only one component, we were accounting for the effects of other components in the reference group. The collective effect of all components was assessed through a log-binomial regression model with all the MetS components taken together after adjusting the other control variables. Table 4 shows the log-binomial regression of CRC on all MetS components together. Taken collectively, high HTN and HG were not significant (*p*-values of 0.536 and 0.752, respectively) in determining CRC status, but IR and low HDL were significant (*p*-values < 0.001 and 0.002). In the presence of all other factors, IR (*p*-value < 0.001) and low HDL (*p*-value = 0.002) were two major components of MetS that were significant in determining CRC status. We also observed that BMI, insurance status, age group, tobacco use, alcohol use, history of CRC and inherited syndromes, history of inflammatory bowel disease, and history of polyps were also significant (*p*-values < 0.05). In the presence of IR and low HDL, high HTN and HG were not significant predictors of CRC. This required us to assess how IR and low HDL impact the likelihood of CRC after adjusting other controls.

Table 5 presents the combined effect of IR and low HDL on CRC. The log-binomial regression analysis of CRC on both IR and low HDL after accounting other variables indicated that patients with both IR and low HDL had significantly higher risk of CRC compared to MetS, as well as those with components other than IR and low HDL. This result was consistent for both the groups of patients younger and older than 65 years. Patients with both IR and low HDL were 2.65 times more likely to have CRC compared to patients with other components, HTN, TG, or obesity (*p*-value < 0.001). Similarly, the risk of CRC among patients with both IR and low HDL components was 2.39 and 2.62 times higher than among patients with components other than IR or low HDL for both age groups, those less than 65 yrs. and those more than 65 yrs., respectively. This clearly indicates that IR and low HDL are the two most important components of MetS, and that patients with IR and low HDL are at higher risk of developing CRC. The area under the curve (AUC, %) of the receiver operating characteristic curve (ROC) also confirmed the importance of both IR and low HDL. Compared to the younger population (<65 yrs.), the older population (65+ yrs.) with both IR and low HDL was at higher risk of CRC.

## 4. Discussion

This study delved into the relationships between MetS and CRC components to assess the necessity for CRC screening among those presenting with MetS and its specific elements. By analyzing the combined and individual impacts of these components, we found that IR and low HDL emerged as the most significant when considered collectively. Individually, each component of MetS, including IR, low HDL, TG, obesity, and HTN, posed significant CRC risks, though confounding was evident due to the presence of multiple components in most patients. To address this problem, we examined all components together, revealing that patients with both IR and low HDL faced a significantly higher risk than those with other components. This pattern persisted across both age groups, including those under and over 65 years.

Our findings aligned with several previous studies that established the relationship between MetS and CRC and indicated increased CRC risk among patients with MetS [7,8,9,10,11,12,13,14,15]. Additionally, IR was identified as a critical factor, likely due to insulin’s role as a growth factor for colonic cells and its mitogenic effect on tumor cells [31]. Supporting this, a study by Komninou et al. highlighted insulin as a key biochemical link between obesity and colon cancer, suggesting that managing hyperinsulinemia could reduce cancer risk [32]. The significance of IR was echoed by other studies [33,34], which discussed its role in colon cancer progression [9]. The impact of low HDL on CRC has been much less studied, but emerging evidence suggests that there is a correlation between lower HDL levels and better prognostic outcomes in various cancers, such as breast and prostate cancer [35,36]. Lastly, limited information is known about the effects of hyperglycemia (HG) and its relation to colorectal neoplasms. Some research supports a link between HG, hypertriglyceridemia, hyperinsulinemia, and CRC through insulin resistance [37,38,39], while other studies report conflicting results [40,41,42]. In our study, HG emerged as a significant individual risk factor, but its collective impact with other MetS components was insignificant. This indicates that the effects of HG on CRC were mediated through components like IR or low HDL.

Our study has several strengths that contribute to its significance in the field of research on colorectal cancer (CRC) and metabolic syndrome (MetS). First, it utilizes a large dataset from the Appalachian region, covering a decade and representing 8.2% of the U.S. population. To the best of our knowledge, this is the first study to comprehensively examine CRC incidence and MetS in this area.

Second, we employed the robust and well-validated SAS statistical package, which enhanced the comparability of our results with those of other studies.

Third, although several studies have explored the relationship between MetS and CRC [7,8,9,10,11,12,13,14,15], few have investigated the link between individual MetS components—other than obesity—and CRC. Our research helps fill this gap by adjusting for multiple risk factors such as age, sex, BMI, insurance status, tobacco and alcohol use, family history of CRC, inherited syndromes, inflammatory bowel disease, polyps, and type II diabetes, all of which are known correlates of CRC [16,22,23,24,25,26,27,28,29]. We analyzed the individual effects of insulin resistance (IR), low HDL cholesterol (HDL), hypertension (HTN), and high glucose (HG) on CRC risk (Table 3). Furthermore, we individually examined the CRC risk associated with each MetS component (Table 2), an area not previously addressed.

Lastly, and most critically, we identified the collective impact of these factors—IR, low HDL, HTN, and HG—on CRC risk. Our findings indicate a higher prevalence of CRC among older individuals, who typically present multiple MetS components, with the average individual having at least three components. This highlights the importance of studying these components’ simultaneous impact on CRC. Notably, this is the first study to research this after controlling for BMI and other significant risk factors (Table 4). We discovered that individuals presenting both IR and low HDL are at a significantly higher risk of CRC compared to those with other components, a result that is consistent across age groups under and over 65 years (Table 5). This pivotal insight could inform future CRC screening strategies, suggesting earlier screening for individuals with IR and low HDL.

However, our analyses also have a few limitations, including their focus on the Appalachian region, potentially limiting broader applicability to other populations. Obviously, the risk rates were higher in our findings, as this region experiences significantly higher CRC rates than the nation [17]. Still, the risk factors we identified reflect the general pattern for the entire U.S. population. The lack of data on socioeconomic status (SES), race, and diet presented further constraints, as these are significant CRC risk factors [22,24,42,43]. Future research should aim to incorporate these variables to enhance understanding of CRC and develop more effective screening and prevention strategies. Moreover, the rising CRC incidence in younger individuals underlines the need for age-specific studies to tailor prevention efforts effectively [44,45].

## 5. Conclusions

In conclusion, metabolic syndrome and its components, namely, insulin resistance, hypertension, low HDL-C, and hypertriglyceridemia, have been identified as significant risk factors for colorectal cancer (CRC). Most notably, when multiple components were present, individuals with IR and low HDL had a significantly higher risk of CRC than individuals with no components or any other combination of components. This elevated risk was consistent across age groups under and over 65 years. Therefore, it is advisable to prioritize these individuals for earlier CRC screening. Future studies may need to examine the CRC risk factors associated with individuals younger than 50 years old and suggest an appropriate screening strategy, as deaths from colorectal cancer among people younger than age 55 have increased by 2% every year from 2007 to 2016 [1], with an even higher rate in the Appalachian region, which suffers from lower CRC screening rates [17,18,19]. Future research studies should define risk factors and adjust other important components such as age, race, diet, and socioeconomic status (SES).

## Figures and Tables

**Table 1:** Descriptive summary.

Variables		Mean or n	SE or %
**Total Sample**		263,023	
**Age (in yrs.)**		59.9 (median = 61)	0.03
**BMI (in Kg/m^2^)**		30.9	0.02
**No. of Criteria Met**		3.6 (median = 4)	0.00
**Colorectal Cancer (CRC)**			
	Yes	3474	1.3
	No	259,549	98.7
**Gender**			
	Female	140,469	53.4
	Male	122,554	46.6
**Weight Category**			
	Obese	124,456	47.9
	Overweight	77,585	29.9
	Others	57,704	22.2
**Age Group**			
	<50 yrs.	68,610	26.1
	50–65 yrs	84,498	32.1
	>=65 yrs.	109,915	41.8
**Insurance**			
	Medicaid or Medicare	14,964	5.7
	Others or unknown	248,059	94.3
**Tobacco Use**			
	Yes	128,469	48.8
	No	134,554	51.2
**Alcohol Use**			
	Yes	31,183	11.9
	No	231,840	88.1
**Family History of CRC or Inherited Syndromes**			
	Yes	17,617	6.7
	No	245,406	93.3
**Inflammatory Bowel Disease**			
	Yes	15,485	5.9
	No	247,538	94.1
**History of Polyps**			
	Yes	21,219	8.1
	No	241,804	91.9
**Type II Diabetes**			
	Yes	96,162	36.6
	No	166,861	63.4
**MetS**			
	Yes	211,510	80.4
	No	51,513	19.6
**IR**			
	Yes	194,400	73.9
	No	68,623	26.1
**HDL**			
	Yes	212,373	80.7
	No	50,650	19.3
**HTN**			
	Yes	211,116	80.3
	No	51,907	19.7
**TG**			
	Yes	204,356	77.7
	No	58,667	22.3
**IR and HDL Only**			
	both IR and HDL but no TG, HTN, or obesity	1812	10.5
	HTN, TG, or obesity but no IR or HDL	15,495	89.5

SE: standard error.

**Table 2:** Risk of CRC among different components of METS.

Components	n	Risk Ratio (RR)	95% CI	Attributable Risk (per 10,000)
**MetS**	3092	1.97	(1.77, 2.19)	72
**IR**	2970	2.08	(1.89, 2.29)	79
**HDL**	3068	1.8	(1.63, 2.00)	64
**HTN**	3071	1.87	(1.69, 2.08)	68
**TG**	3008	1.85	(1.68, 2.04)	68

**Table 3:** Log-binomial regression analyses of CRC on MetS and its individual components taken separately.

		CI		
Symptom	RR	LL	UL	*p*-Value
**MetS**	1.28	1.14	1.42	<0.001
**IR**	1.60	1.45	1.76	<0.001
**HDL**	1.29	1.16	1.44	<0.001
**HTN**	1.13	1.01	1.26	<0.001
**TG**	1.17	1.06	1.30	<0.001

**Table 4:** Log-Binomial regression of CRC on all MetS components after adjusting other control variables.

		CI		
Variables	RR	LL	UL	*p*-Value
**IR (Reference = No IR)**	1.55	1.40	1.71	<0.0001
**HTN (Reference = No HTN)**	1.04	0.93	1.16	0.536
**HDL (Reference = No HDL)**	1.24	1.08	1.42	0.002
**TG (Reference = No TG)**	0.98	0.86	1.12	0.752
**BMI**	1.08	1.06	1.10	<0.0001
**Gender (Male; Reference = Female)**	1.02	0.95	1.09	0.563
**Insurance (Reference = Others)**	1.46	1.26	1.70	<0.0001
**Age Group 50-65 yrs (Reference = <50 yrs)**	1.93	1.69	2.21	<0.0001
**Age Group 65 yrs. and above (Reference = <50 yrs)**	3.21	2.81	3.68	<0.0001
**Smoke (Reference = No History of Smoking)**	1.22	1.14	1.31	<0.0001
**Alcohol Use (Reference = No Alcohol Use)**	1.32	1.20	1.45	<0.0001
**History of CRC or Inherited Diseases** **(Reference = No History of CRC or Inherited Diseases)**	3.64	3.31	4.01	<0.0001
**History of Inflammatory Bowel Disease** **(Reference = No History of Inflammatory Bowel Disease)**	1.89	1.71	2.09	<0.0001
**History of Polyps (Reference = No History of Polyps)**	1.75	1.59	1.93	<0.0001

**Table 5:** Log-binomial regression analyses of CRC on IR and HDL combinations.

		CI			
Symptom	RR	LL	UL	*p*-Value	ROC-AUC (%)
**MetS**	1.28	1.14	1.42	<0.001	72.6
**IR+HDL Only**					
Both IR and HDL	2.65	1.68	4.20	<0.001	79.5
HTN or TG or Obese but no IR or HDL	Reference				
**Among Individuals <65 yrs. Old**					
**IR+HDL**					
Both IR and HDL	2.39	1.35	4.23	<0.001	76.9
HTN or TG or Obese but no IR or HDL	Reference				
**Among 65-or-Older Age Group**					
**IR+HDL Only**					
Both IR and HDL	2.67	1.25	5.69	<0.001	71.1
HTN or TG or Obese but no IR or HDL	Reference				

Age, sex, BMI, insurance information, smoking, alcohol consumption, family history of CRC or inherited syndromes, history of inflammatory bowel disease, and history of polyps were controlled. Note: For MetS analysis, BMI was not controlled, as obesity is one of the components of MetS. RR = Odds Ratio. LL = Lower Limit. UL = Upper Limit. CI = 95% Confidence Interval. ROC-AUC—degree of separability between CRC groups. *p*-value < 0.05 is significant.

## Data Availability

Data will be provided to investigators actively engaged in CRC investigation for verifiable query.
